# Serological diagnosis of fasciolosis (*Fasciola hepatica*) in humans, cattle, and sheep: a meta-analysis

**DOI:** 10.3389/fvets.2023.1252454

**Published:** 2023-08-31

**Authors:** Guilherme Drescher, Tassia Cristina Bello de Vasconcelos, Vínicius Silva Belo, Mariane Marques da Guarda Pinto, Jaqueline de Oliveira Rosa, Luis Gustavo Morello, Fabiano Borges Figueiredo

**Affiliations:** ^1^Cellular Biology Laboratory, Carlos Chagas Institute, Oswaldo Cruz Foundation (FIOCRUZ-PR), Curitiba, Brazil; ^2^Auditora Fiscal Federal Agropecuária do Ministério da Agricultura Pecuária e Abastecimento (MAPA), Curitiba, Brazil; ^3^Programa de Pós-Graduação Ciências da Saúde, Universidade Federal de São João Del Rei, Divinópolis, Brazil; ^4^Trypanosomatid Molecular Biology Laboratory, Carlos Chagas Institute, Oswaldo Cruz Foundation (FIOCRUZ-PR), Curitiba, Brazil; ^5^Laboratory for Applied Science and Technology in Health, Carlos Chagas Institute, Oswaldo Cruz Foundation (FIOCRUZ-PR), Curitiba, Brazil; ^6^Parana Institute of Molecular Biology, Curitiba, Brazil

**Keywords:** meta-analysis, *Fasciola hepatica*, native antigen, recombinant antigen, human and animals

## Abstract

*Fasciola hepatica* can cause problems in both animals and humans. Fasciolosis can be diagnosed through the indirect ELISA immunodiagnostic test. Serological diagnosis of *Fasciola* is based on recombinant antigens secreted by this worm. We used PubMed and Google Scholar databases to review the published literature on ‘antigens with immunogenic potential’ used in serological tests to identify antibodies against *F. hepatica* in humans, cattle, and sheep. Studies that investigated diagnostic tests with common reference standards were included in the sensitivity and/or specificity bivariate meta-analysis. In the quality and susceptibility to bias analysis of the 33 included studies, 26 fulfilled at least six (75%) of the eight QUADAS criteria and were considered good-quality papers. We found that most of the studies used native excretory-secretory antigens and recombinant cathepsin in ELISA tests for serological diagnosis of fascioliasis in humans, cattle, and sheep. The meta-analysis revealed that all antigens demonstrated good accuracy. The best results in terms of sensitivity [0.931–2.5% confidence interval (CI) and 0.985–97.5% CI] and specificity (0.959–2.5% CI and 0.997–97.5% CI) were found in human *Fh*ES. *Fh*rCL-1, *Fh*ES, and *Fh*rSAP-2 antigens gave the best results for the serum diagnosis of human and animal fasciolosis.

## 1. Introduction

In recent years, there has been a high level of concern worldwide about the incidence of foodborne trematode (FBT) infections. The parasites responsible for this infection include *Fasciola hepatica* and *F. gigantica* flatworms, which pose a major problem for animals and humans (1). They have a complex life cycle, using Lymnaeidae snails as an intermediate host, a carrier (aquatic plants), and a final mammalian host (cattle, sheep, or even humans). In humans, this parasitosis is acknowledged to be a (re-)emerging disease in several countries that has spread in close association with climatic conditions (2). Almost 80 species of intestinal flukes infect humans and animals worldwide (3, 4). However, in South America, only *F. hepatica* has been identified in humans and cattle (3). Fasciolosis is considered an important endemic disease in this part of the American continent (2, 5–7).

Bovine fasciolosis occurs on every continent except Antarctica, and over 700 million animals are estimated to be at risk of infection. The cost to the farming and industry of *F. hepatica* infection in cattle is estimated to be over 3 billion USD per year worldwide (8, 9). This cost is largely unquantified at national or regional levels, and it has been reported that fluke affects milk yield and carcass composition, prolonging the time required to reach slaughter weight (10–12). It is therefore important to develop methods to identify liver fluke infections.

The gold standard for diagnosing trematode infection involves examining fecal eggs, which can be performed through ether concentration, sedimentation techniques, or the Kato–Katz method. In the case of visceral inspection, the presence of worms in the liver can also be used (13–16). FBT infections are usually diagnosed through imaging, immunodiagnostic, and molecular techniques (humans), as well as parasitological methods (animals). Immunodiagnostic testing commonly includes the indirect enzyme-linked immunosorbent assay (ELISA), indirect hemagglutination, indirect fluorescent antibody test, and intradermal testing. For serological diagnosis, Fasciola ELISA tests use different antigens for antibody detection in humans, sheep, and cattle. These antigens include a series of proteolytic enzymes, such as proteases and glutathione S-transferases, which the parasite uses to survive in the host body (17–19). These enzymes have been implicated in several aspects of helminth development (18).

Initially, serologic tests used to diagnose fasciolosis relied on a somatic antigen (SA) obtained from adult flukes collected from the bile ducts of cows at slaughterhouses (20). This method was less specific than other, more modern tests. Subsequently, ELISA tests were developed to detect antibodies in human and animal sera, using excretory-secretory (ES) *Fasciola* sp. antigens. These antigens, which are excreted and secreted by liver fluke, are immunogenic and can modulate host immune responses. More recent testing methods have used recombinant *Fasciola* antigens and ELISA tests have been developed to detect antibodies in human and animal sera (21). Standardization in recombinant protein preparation is important in these cases to increase production. Recombinant antigen production is also more cost-effective than ES preparation (21, 22).

In recent years, a wide range of targeted *F. hepatica* genes has been chosen, cloned, and produced in various expression host systems (bacteria and yeast) using different expression conditions to achieve an ideal diagnostic test for human fasciolosis. Recombinant saposin-like protein 2 antigen (rSAP-2) (23–25), recombinant leucine aminopeptidase (rLAP) (26), recombinant glutathione S-transferase (27, 28), and recombinant cathepsin L1 (rCL-1) (21, 22, 29) are the most immunodominant antigens. Trematodes secrete a large family of cysteine proteases (30) that include cathepsin L1 (CL-1), cathepsin L2 (CL-2), cathepsin L3 (CL-3), and cathepsin L5 (CL-5) (30, 31).

Proteomic analysis of *F. hepatica* secretions identified cathepsin L1 enzymes as the main components involved in virulence (32). They can cleave several host substrates in the host blood for parasite feeding, migration through host tissues, formation of eggshells, and excystment (30). Cathepsin proteins can be found in juvenile and adult liver flukes (18, 30). Cathepsin L proteases are the most predominant components of ES antigens, which are used globally as immunodiagnostic tools for diagnosing liver fluke infections in humans and animals (33).

Understanding the role principal proteases involved in *F. hepatica* host invasion is the first step toward developing serologic diagnostic tests for humans and animals. In the case of humans, lateral flow immunoassay (LFI) tests have already been developed for fasciolosis (33). ELISA and LFI tests use *F. hepatica* proteins as antigens to identify antibodies in human and animal sera or feces (22, 29, 34–36). We therefore conducted a meta-analysis of the literature using the terms “antigens (native and recombinant) with immunogenic potential” used in serological tests to identify antibodies against *F. hepatica* in humans, cattle, and sheep. Our principal aims were to evaluate the quality of the selected papers and then perform a meta-analysis to identify the best antigen options.

## 2. Materials and methods

### 2.1. Information sources and selection of studies

For the systematic review (SR), the Google Scholar and PubMed databases were used up to November 2022. No restrictions were placed on study publication dates. Chart 1 shows the search strategy, index terms, and inclusion and exclusion criteria used. The references of the chosen publications were also analyzed to identify additional papers. The protocol was included in the PROSPERO registry (ID:412565).

**CHART 1:** Search strategies and inclusion and exclusion criteria applied in the SR of peptides with immunogenic potential used in serological tests to identify antibodies against *F. hepatica* in humans and ruminants.

**Search strategy:** **PubMed**: (*Fasciola* OR *Fasciola hepatica* OR Fasciolosis) AND (Humans OR Ruminant OR Cattle OR *Bos* OR Bovine OR Sheep, domestic OR *Ovis*) AND (Diagnostic test OR Enzyme-Linked Immunosorbent Assay OR ELISA OR Peptides OR Recombinant antigen OR Recombinant Proteins OR Validation Studies) **Google Scholar**: (*Fasciola* OR Fasciolosis) (Human OR Ruminant OR Cattle OR *Bos* OR Bovine OR Sheep, domestic OR *Ovis*) (Diagnostic test OR Enzyme-Linked Immunosorbent Assay OR ELISA OR Serological test OR peptides OR recombinant antigen OR Validation Studies)
**Inclusion criteria:** Studies that followed the Population, Interventions, Comparison, Outcomes, and Study (PICOS) design criteria (37): Population: humans, ruminant cattle, and sheep Interventions (index tests): *Fasciola* serological indirect diagnostic test Comparator: gold standard technique of parasite identification (fecal egg detection and presence of *Fasciola* in the liver inspection *post-mortem*) Outcome: studies that have reported diagnostic sensitivity and specificity Study design: field validation study designs for *Fasciola* diagnostic by rapid tests
**Exclusion criteria:** Publications covering the following topics were excluded: Studies on other animal species Direct ELISA (serum antigen and coproantigen for fasciolosis) Indirect ELISA (*Fasciola* milk indirect ELISA diagnostic test) Molecular test (based on DNA detection) Studies in *Fasciola gigantica* and *Fasciola magna*

### 2.2. Evaluation of limitations and potential bias

The Quality Assessment of Diagnostic Accuracy Studies (QUADAS) tool was used to evaluate the publication quality (38, 39). This tool contains 14 criteria, with eight considered applicable to this study (see Chart 2). Three additional questions from the Standards for Reporting of Diagnostic Accuracy (STARD) checklist (40) were included to provide essential information when evaluating epidemiological studies and methods, as suggested by several authors (38, 39). Therefore, the selected articles were read and analyzed using the combination of eight QUADAS and three STARD criteria.

**CHART 2:** QUADAS criteria for assessing the quality of the studies included in this SR on peptides or recombinant proteins with immunogenic potential used in serological tests to identify antibodies against *F. hepatica* in humans and ruminants.

Was the spectrum of samples representative of the specimen that will receive the test in practice?
Were selection criteria clearly described?
Is the period between the reference standard and index test short enough to be reasonably sure that the target condition did not change between the two tests?
Did samples receive the same reference standard regardless of the index test result?
Was the reference standard independent of the index test (i.e., the index test was not part of the reference standard)?
Was the execution of the index test described in sufficient detail to permit replication of the test?
Were the same clinical data available when test results were interpreted as would be available when the test is used in practice?
Were withdrawals from the study explained?

The QUADAS and STARD criteria are presented in Charts 2, 3, respectively:

**CHART 3:** STARD criteria for assessing the quality of the studies included in this SR on peptides or recombinant proteins with immunogenic potential used in serological tests to identify antibodies against *F. hepatica* in humans and ruminants.

Is the sampling process described?
Are sensitivity and specificity results reported with their respective confidence intervals (CIs)?
Are clinical and demographic characteristics of the animal population reported (e.g., age, sex, spectrum of presenting symptoms, comorbidity, current treatments—among others)?

For both analyses, the responses to the questions were categorized as “yes,” “no,” or “unclear.” The quality analysis method followed that described by De Oliveira et al. (38), with some modifications. In the QUADAS analysis, studies that met four to five criteria (corresponding to 50–60% “yes” answers)—were considered to be of “regular” or “good” quality. A cutoff point of 75%, where at least six criteria were met, was used to define a “good” quality study. For STARD, good-quality studies were considered those meeting all three STARD criteria. The quality criteria were applied independently by two researchers—disagreements were resolved by a third reviewer who participated in the analysis of the specific criteria in question.

### 2.3. Meta-analysis

The articles were organized into 13 groups according to host species and antigen parasite characteristics to verify the possibility of performing a meta-analysis. This included six human groups: humans using *F. hepatica* excretory-secretory proteins (*Fh*ES), human somatic antigens (*Fh*SA), human recombinant ferritin (*Fh*rFtn-1), human tegument-associated protein (*Fh*TP16.5), human recombinant saposin (*Fh*rSAP-2) and recombinant cathepsin (*Fh*rCL-1); two cattle groups: cattle *Fh*ES and cattle *Fh*rCL-1; and five sheep groups: sheep *Fh*ES, sheep *Fh*SA, sheep *Fh*rCL-1, sheep fatty acid binding recombinant protein (*Fh*rFAB), and sheep glutathione S-transferase recombinant protein (*Fh*rGST). Meta-analyses were performed only if there were more than two eligible studies in each group.

The bivariate binomial random effects model of Chu and Cole (41) was applied to the meta-analysis. Sensitivity and specificity were jointly modeled with the estimates from each study. It was assumed they varied but came from a common underlying distribution with an unstructured between-study covariance matrix (42). All models were fitted without covariates. The hierarchical summary receiver operating characteristic (HSROC) parameters were used to draw the summary receiver operating characteristic (SROC) plots. The percentage of the study weights was calculated using the methodology of Burke et al. (43). Sensitivity analyses were conducted by removing studies assessed as having low quality. This did not result in any significant changes in the pattern of the results obtained.

Considering the number of studies, heterogeneity was assessed by visual inspection of forest plots and confidence intervals (CI) of sensitivity and specificity of primary studies. The analyses were performed in the MetaDTA program (44). Chart 4 lists the papers used for the meta-analysis in each group.

**CHART 4:** Articles included in meta-analysis performance in relation to sensitivity, specificity, and diagnostic odds ratio by groups, related to host species and antigen parasite characteristics—human *Fasciola hepatica* excretory-secretory (*Fh*ES) proteins and human *Fasciola hepatica* Somatic Antigen (*Fh*SA); cattle *Fh*ES and cattle *Fasciola hepatica* recombinant Cathepsin L-1 (*Fh*rCL-1); and sheep *Fh*ES.

**Paper group**	**References**
Human *Fh*ES	Aguayo et al. (27); Mirzadeh et al. (25); Gottstein et al. (24); Morales and Espinosa (45); Cornejo et al. (46); Espinoza et al. (47); Rokni et al. (48); Figueroa-Santiago et al. (23); Carnevale et al. (49); Córdova et al. (50)
Human *Fh*SA	Shafiei et al. (51); Rahimi et al. (52); Maher et al. (53)
Cattle *Fh*ES	Mufti et al. (54); Kuerpick et al. (22); Şimsek et al. (55); Salimi-Bejestani et al. (56); Cornelissen et al. (35); Hillyer et al. (57)
Cattle *Fh*rCL-1	Martínez-Sernández et al. (29); Kuerpick et al. (22); Cornelissen et al. (36)
Sheep *Fh*ES	Cornelissen et al. (36); Heidari et al. (58); Kooshan et al. (59); Mezo et al. (60); Hillyer et al. (57)

## 3. Results

**Figure 1:**
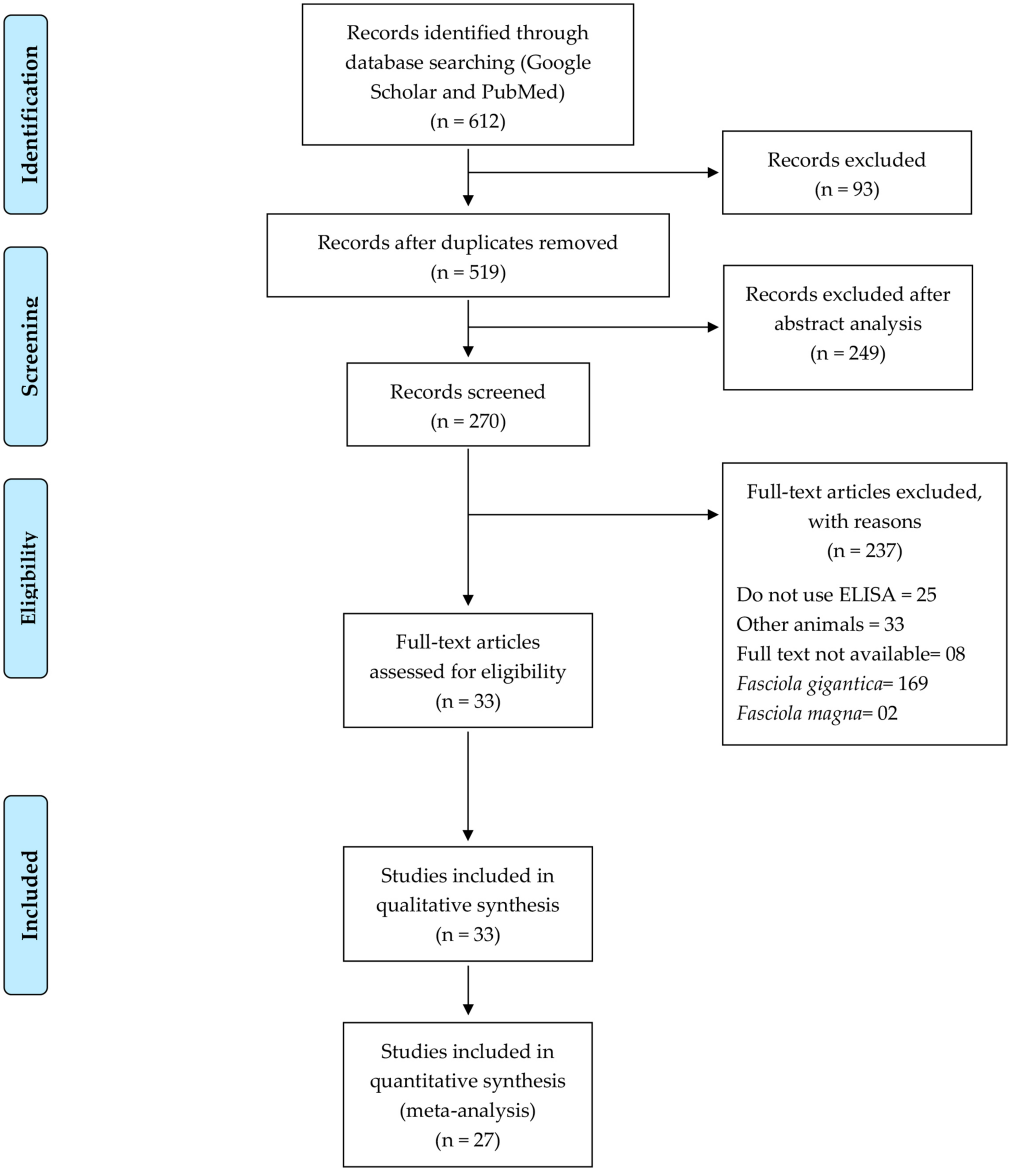
Flow diagram of the study selection process (37).

The first search yielded 1,073 from PubMed and 1,436 articles from Google Scholar. After reviewing the titles, 612 studies met the inclusion criteria. These papers were analyzed according to the search strategy, and after removing duplicates (93), 519 titles remained, 249 of which were excluded after reading the abstract, leaving 270 papers. Of these, 169 were disregarded as they discussed *F. gigantica,* and another two studies were excluded for discussing *F. magna*. Thirty-three papers discussed other animal species, and eight publications could not be accessed online. Of the remaining 58 full-text articles assessed for eligibility, 25 were excluded for not using an ELISA test. Accordingly, 33 studies were included in the qualitative synthesis, and 27 papers were included in the meta-analysis (Figure 1).

### 3.1. Quality assessment of included studies

Twenty-six articles met at least six (75%) of the eight QUADAS criteria and were considered to be good-quality papers. Six articles met 4–5/8 criteria (50–60%) and were categorized as regular studies. Only one reached 4/8 QUADAS criteria, suggesting that it was of lower quality.

For STARD, only two studies met all three criteria and were considered good-quality studies. Fifteen were categorized as having met two STARD criteria, and 16 studies were classified as having met just one STARD criterion (Supplementary Charts S1, S2).

### 3.2. Antigen target from ELISA

The meta-analysis considered seven native and recombinant antigens used for serological diagnosis of fasciolosis in different animals, including humans. The selected studies showed that the most common antigen used in ELISA tests was *Fh*ES. The second most common ones were recombinant cathepsin L1 and recombinant saposin. Other proteins also appeared in this review, such as native SAs of *F. hepatica* as well as other recombinant antigens, namely recombinant ferritin, fatty acid binding recombinant protein, and glutathione S-transferase recombinant protein.

Most studies focused on humans (20 papers) using *Fh*ES, *Fh*rCL-1, and other antigens. Nine articles discussed the *Fh*ES antigens for serological diagnosis of human fasciolosis. Several tests targeting antibodies against the previously described *Fh*rCL-1 (*n* = 3) in humans and cattle have been developed. Other recombinant proteins (*Fh*rFtn-1, *Fh*rFAB, and *Fh*rGST) were also used for serological diagnosis of fasciolosis in humans, cattle and sheep.

The highest sensitivities and specificities for diagnosing fasciolosis were obtained using human samples. Serological ELISA tests that used *Fh*ES, *Fh*rCL-1, *Fh*SA, and *Fh*rSAP-2 proteins gave similar results for humans, cattle, and sheep. False positives were reported for all antigens (native and recombinant) and were not linked to any particular parasitic infection. No cross-reaction was reported for any antigen (native or recombinant) for any of the analyzed species.

### 3.3. Meta-analysis results

Of the 13 groups, only five contained more than two studies that could be used for meta-analysis. These were human *Fh*SA and *Fh*ES, cattle *Fh*ES and *Fh*rCL-1, and sheep *Fh*ES (Supplementary Chart S3).

**Figure 2:**
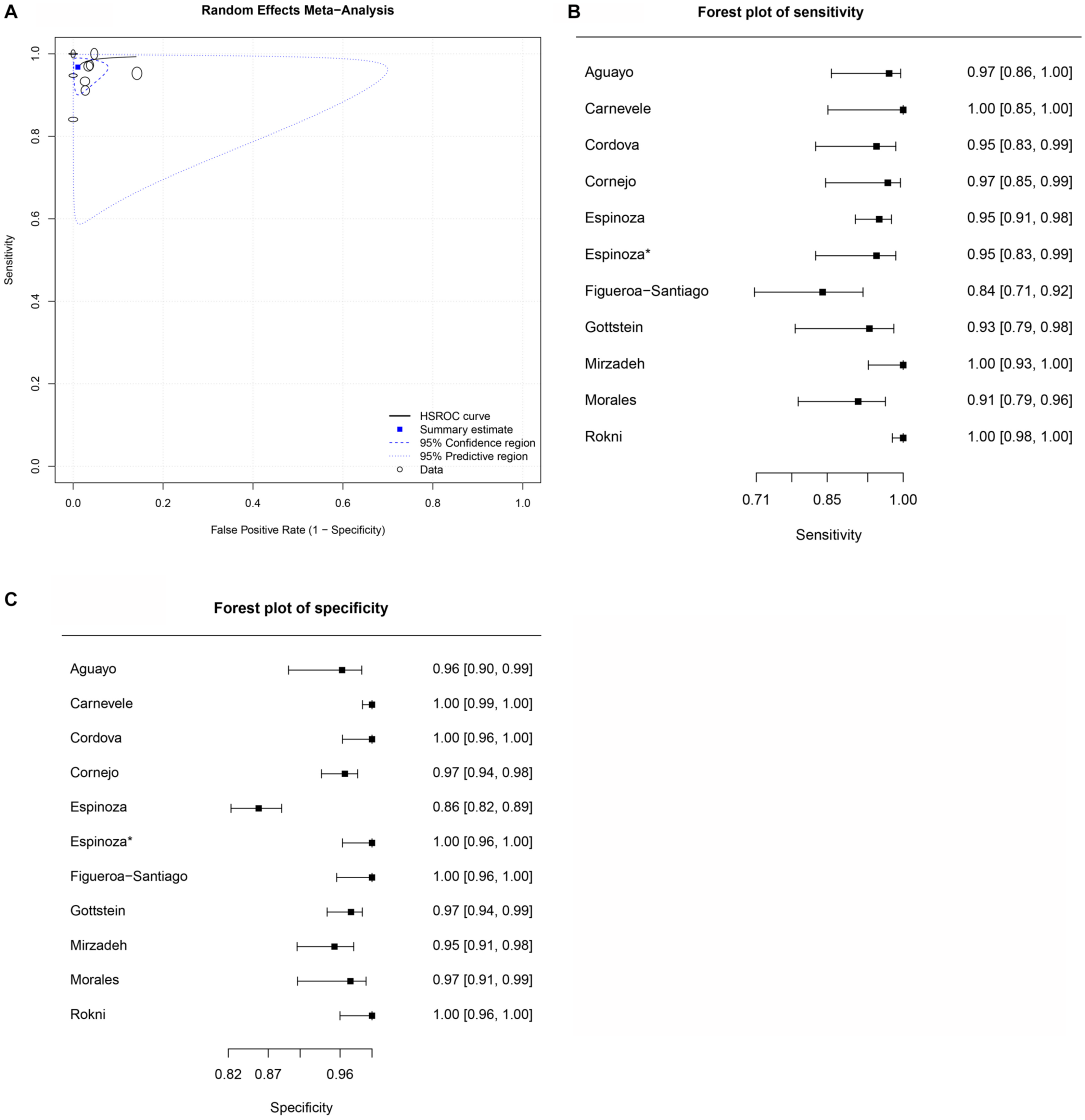
Forest plots for the human *Fh*ES proteins paper group. **(A)** Random effects meta-analysis for human excretory-secretory proteins paper group. The size of the circles determines the weight of the study. **(B)** Forest plot sensitivity for human excretory-secretory proteins paper group. *This corresponds to a second approach by Espinosa et al. (47), in the same article, in field research. **(C)** Forest plot specificity for human excretory-secretory proteins paper group.

**Figure 3:**
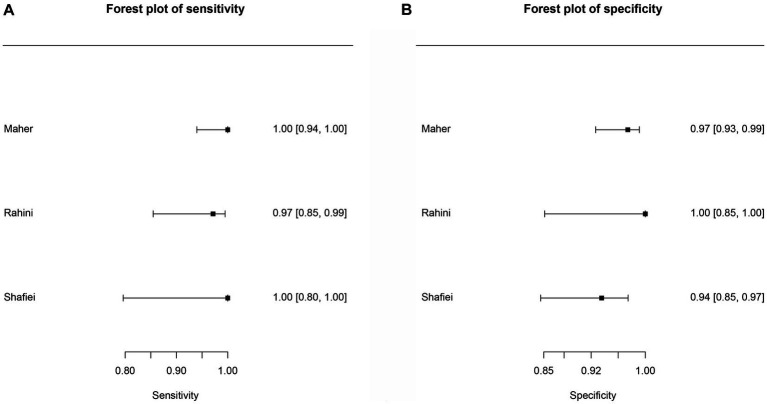
Forest plots for the human *Fh*SA proteins paper group. **(A)** Forest plot sensitivity for human somatic antigens paper group. **(B)** Forest plot specificity for human somatic antigens paper group.

**Figure 4:**
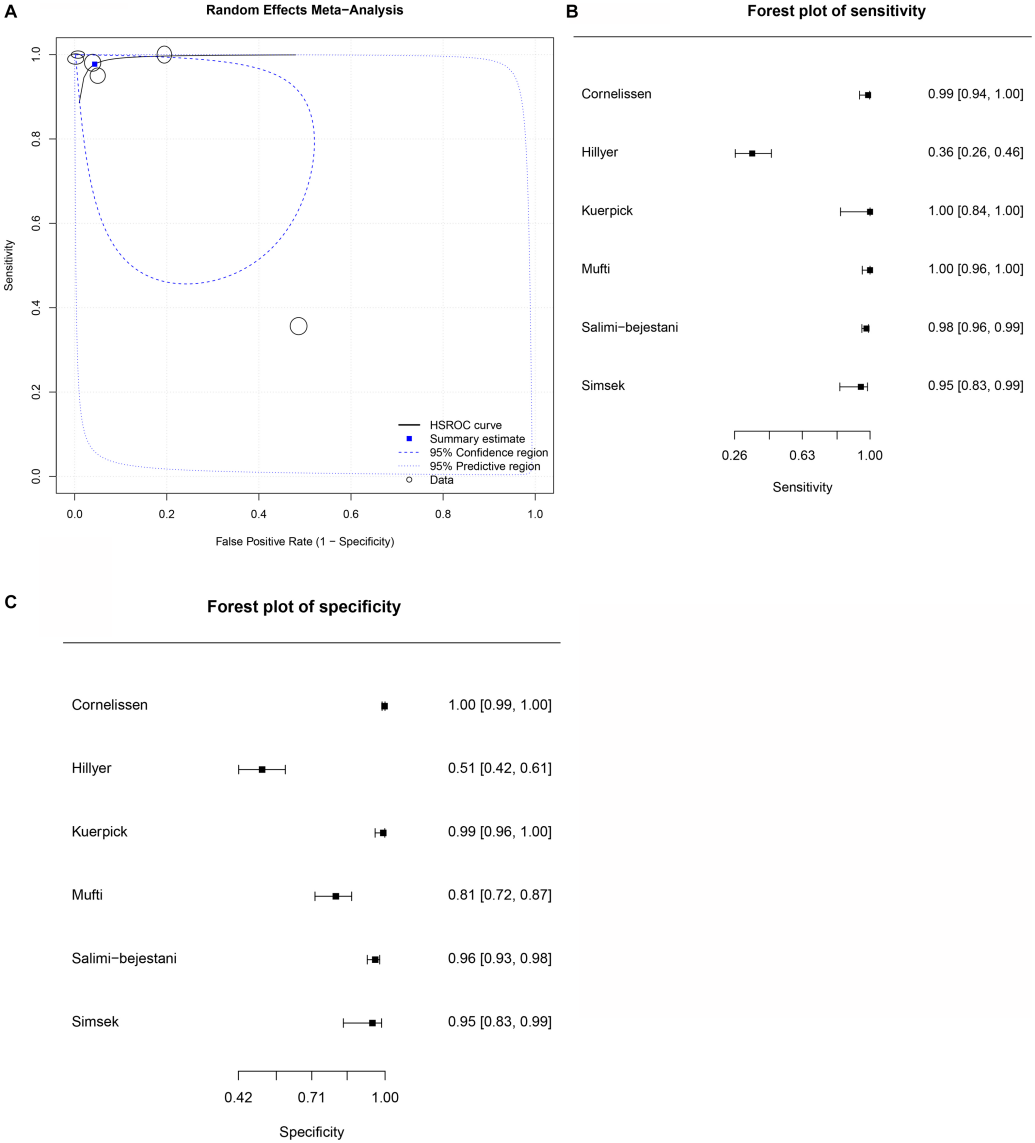
Forest plots for the cattle *Fh*ES proteins paper group. **(A)** Random effects meta-analysis for cattle excretory-secretory proteins paper group. The size of the circles determines the weight of the study. **(B)** Forest plot sensitivity for cattle excretory-secretory paper group. **(C)** Forest plot specificity for cattle excretory-secretory proteins paper group.

**Figure 5:**
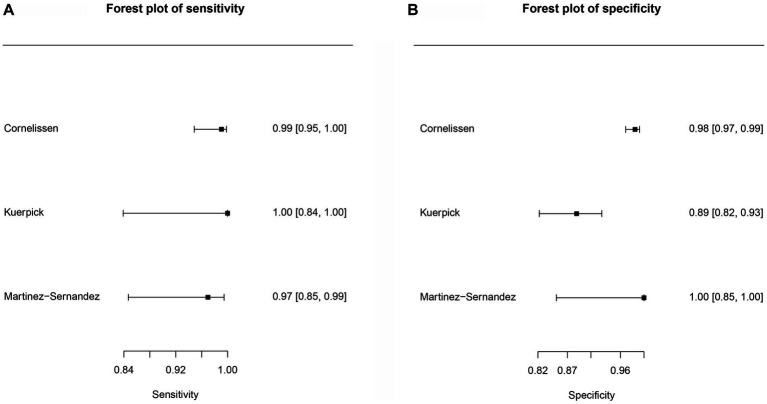
Forest plots for the cattle *Fh*rCL-1 protein paper group. **(A)** Forest plot sensitivity for cattle recombinant cathepsin antigens paper group. **(B)** Forest plot specificity for cattle recombinant cathepsin paper group.

Tables 1–5 show the sensitivity, specificity, and diagnostic odds ratio (DOR) values combined in the bivariate meta-analyses by paper group. For each group, SROC plots, forest plots of sensitivity, and forest plots of specificity (Figures 2–5) are presented, except for the *Fh*SA and cattle *Fh*rCL-1 groups, for which no SROC plots could be produced, as only they only contained three papers each.

**Table 1:** Sensitivity, specificity, and diagnostic odds ratio values combined in the bivariate meta-analyses for human *Fh*ES proteins paper group.

Parameter	Estimate	2.5% CI	97.5% CI
Sensitivity	0.968	0.931	0.985
Specificity	0.989	0.959	0.997
Diagnostic odds ratio	2819.090	553.512	14357.904
Positive likelihood ratio	88.000	22.710	328.330
Negative likelihood ratio	0.030	0.070	0.020

CI, confidence interval.

**Table 2:** Sensitivity, specificity, and diagnostic odds ratio values combined in the bivariate meta-analyses for human *Fh*SA proteins paper group.

Parameter	Estimate	2.5% CI	97.5% CI
Sensitivity	0.991	0.938	0.999
Specificity	0.965	0.928	0.983
Diagnostic odds ratio	3005.286	364.935	24748.890
Positive likelihood ratio	28.310	13.030	56.760
Negative likelihood ratio	0.010	0.070	0

CI, confidence interval.

**Table 3:** Sensitivity, specificity, and diagnostic odds ratio values combined in the bivariate meta-analyses for cattle *Fh*ES proteins paper group.

Parameter	Estimate	2.5% CI	97.5% CI
Sensitivity	0.977	0.844	0.997
Specificity	0.956	0.806	0.991
Diagnostic odds ratio	946.206	32.704	27376.365
Positive likelihood ratio	22.200	4.350	110.780
Negative likelihood ratio	0.020	0.190	0

CI, confidence interval.

**Table 4:** Sensitivity, specificity, and diagnostic odds ratio values combined in the bivariate meta-analyses for cattle *Fh*rCL-1 proteins paper group.

Parameter	Estimate	2.5% CI	97.5% CI
Sensitivity	0.991	0.925	0.999
Specificity	0.973	0.871	0.995
Diagnostic odds ratio	3822.455	338.078	43218.264
Positive likelihood ratio	36.700	7.170	199.8
Likelihood ratio negative	0.010	0.090	0

CI, confidence interval.

**Table 5:** Sensitivity, specificity, and diagnostic odds ratio values combined in the bivariate meta-analyses for sheep *Fh*ES proteins paper group.

Parameter	Estimate	2.5% CI	97.5% CI
Sensitivity	0.982	0.925	0.996
Specificity	0.981	0.639	0.999
Diagnostic odds ratio	2827.480	57.583	138836.742
Positive likelihood ratio	51.680	2.560	996.000
Negative likelihood ratio	0.020	0.120	0

CI, confidence interval.

#### 3.3.1. Diagnostic accuracy of antigens selected in meta-analysis

Ten studies were included for the human *Fh*ES protein group (Figures 2B,C). Sensitivity estimates for the *Fh*ES group were high and similar across the analyzed articles (0.968), and the 2.5% CI and (97.5%) values were 0.931 and 0.985, respectively. For specificity estimates, the 2.5% CI and 97.5% CI were 0.989, 0.959, and 0.997, respectively. Table 1 shows the DOR. Two studies from the human *Fh*ES antigen group showed 100% sensitivity and specificity with the ELISA test. All papers that discussed the *Fh*ES antigen and ELISA tests showed a high level of sensitivity, and four articles had 100% sensitivity. In one paper, the specificity was lower than 90%. Four studies showed 100% specificity when using the *Fh*ES antigen for serum diagnosis of *F. hepatica* in humans (Figures 2B,C). The estimated positive summary Likelihood Ratio for this group was 88.000, which stands as the highest value among the analyzed antigens in the meta-analysis (Table 1).

Figure 2 summarizes the overall diagnostic accuracy of the human *Fh*ES protein group. The HSROC curve had a curvilinear shape (Figure 2A), indicating similarity among the papers included in the meta-analysis, with circles showing a similar format. The SROC point is located near the upper left corner of the curve.

For the human *Fh*SA protein group (human *Fh*SA), three studies were included in the meta-analysis (Figure 3). In this case, no SROC plots were not produced. The sensitivity estimates for the human *Fh*SA group were 0.991, and the 2.5% CI and 97.5% CI values were 0.938 and 0.999, respectively. The 2.5% CI and 97.5% CI values for specificity estimates were 0.965, 0.928, and 0.983, respectively. The DOR is shown in Table 2. Among the studies encompassed in the analysis, the groups focusing on the human *Fh*SA protein and the cattle *Fh*rCL-1 protein exhibited the most minimal negative summary Likelihood Ratio values, which suggests a favorable likelihood of accurate negative diagnosis (Tables 2, 4).

Six studies were included in the cattle *Fh*ES protein group (Figures 4B,C). Figure 4A provides an overview of the overall diagnostic accuracy of the cattle *Fh*ES protein group. The summary HSROC curve was not curvilinear, suggesting a heterogeneous distribution between papers. Circles show a similar format. The sensitivity estimates for the cattle *Fh*ES group were 0.977, and the 2.5% CI and 97.5% CI values were 0.844 and 0.997, respectively. The 2.5% CI and 97.5% CI values for specificity estimates were 0.956, 0.806, and 0.991, respectively. Two papers that used the *Fh*ES antigen for serum diagnosis of *F. hepatica* in cattle showed a sensitivity of 100%, and the specificity varied between 85 and 99% (Figures 4B,C). Four studies presented more variation in sensitivity and specificity. Table 3 shows the DOR. The cattle *Fh*ES protein group demonstrated positive and negative summary Likelihood Ratio values of 22.200 and 0.020, respectively (Table 3).

Three studies were included (Figure 5) for the cattle *Fh*rCL-1 protein group. In this case, no ROC curves were produced. The sensitivity estimate for the *Fh*rCL-1 group was 0.991, and the 2.5% CI and 97.5% CI values were 0.925 and 0.999, respectively. For the specificity estimate, the 2.5% CI and 97.5% CI values were 0.973, 0.871, and 0.995, respectively. DOR can be observed in Table 4.

**Figure 6:**
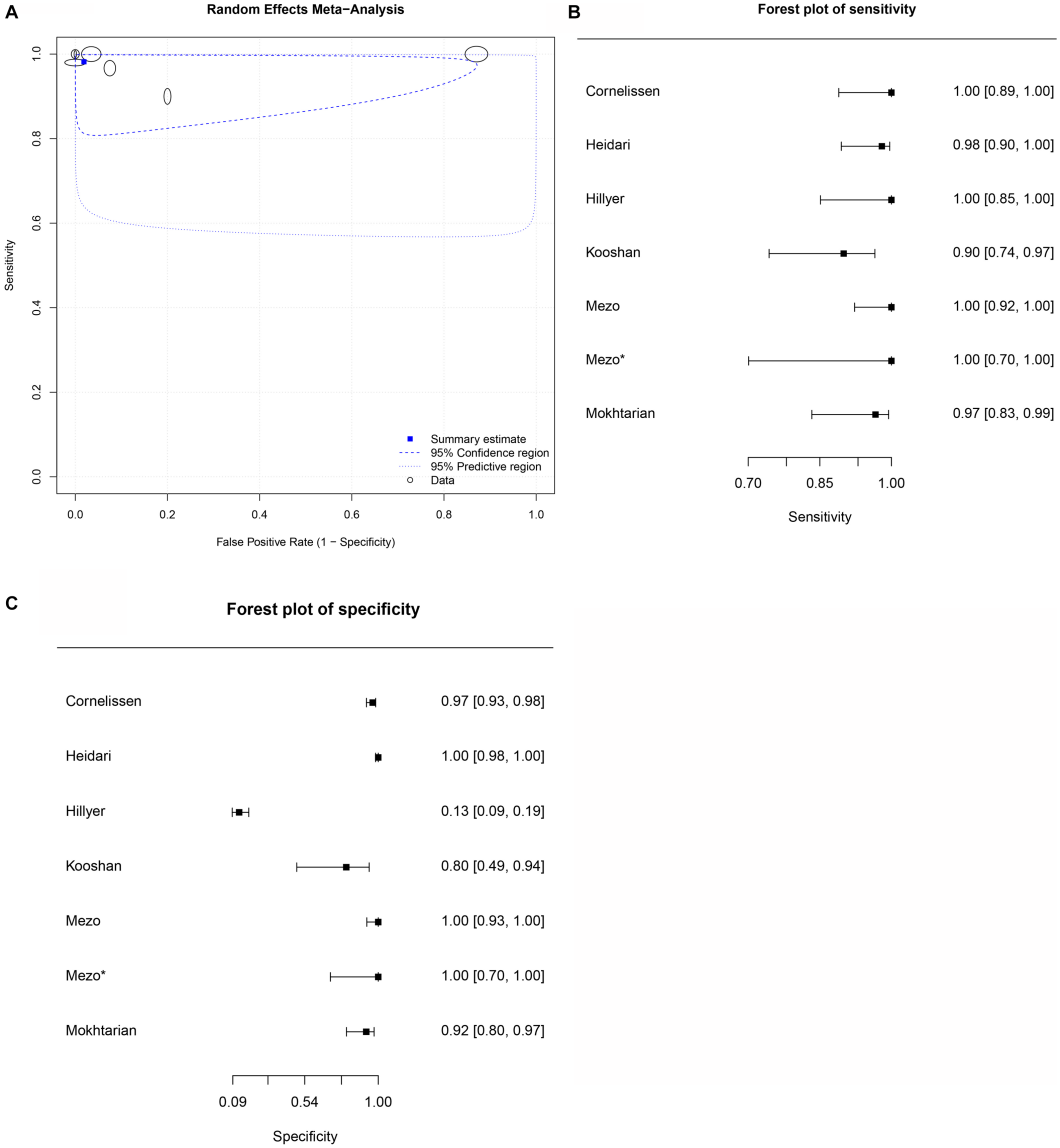
Forest plots for the sheep *Fh*ES proteins paper group. **(A)** Random effects meta-analysis for sheep excretory-secretory proteins paper group. The size of the circles determines the weight of the study. **(B)** Forest plot sensitivity for sheep excretory-secretory proteins paper group. *This corresponds to a second approach by Mezo et al. (60), in the same article, in field research. **(C)** Forest plot specificity for sheep excretory-secretory proteins paper group.

Seven studies were included for the sheep *Fh*ES protein group (Figures 6B,C); the overall diagnostic accuracy is summarized in Figure 6A. The summary HSROC curve was curvilinear, suggesting a homogeneous distribution between papers in the meta-analysis, with circles showing similar formats. The sensitivity estimates for the *Fh*ES group were 0.982, and the 2.5% CI and 97.5% CI values were 0.925 and 0.996, respectively. The 2.5% CI and 97.5% CI values for specificity estimates were 0.981, 0.639, and 0.999, respectively. Table 5 shows the DOR. According to the DOR results, the papers that used *Fh*ES antigens for serological diagnosis of fasciolosis in sheep showed similar results to those of other species. Table 5 presents the positive and negative summary Likelihood Ratio values as 51.680 and 0.020, respectively, further emphasizing the elevated accuracy of the antigen.

Within the five selected groups, the ES antigens of *F. hepatica* were the most extensively studied. Despite the sheep *Fh*ES group having just one study and the lowest specificity, the overall results were good. This group displayed a large variation in terms of specificity. The sheep *Fh*ES group presented similar results between studies as other species.

In general, the reviewed articles gave consistent results, with some studies indicating similarities between serological ELISA tests using different antigens and coprological detection of *F. hepatica* eggs in human, cattle, and sheep feces. Among the analyzed antigens, the cattle *Fh*rCL-1 exhibited homogeneous results, with good accuracy for the serological diagnosis of fasciolosis using ELISA tests. Other native antigens included in the meta-analysis were the human *Fh*SA antigen group, which showed low variation in sensitivity and specificity compared to the identification of *F. hepatica* eggs in human and cattle feces. For the sheep *Fh*ES antigen group, the random effects meta-analysis was more heterogeneous than other native and recombinant antigen groups. Based on the DOR results, studies that used human and cattle *Fh*ES as antigens for serological diagnosis of fasciolosis in humans, cattle, and sheep showed good results compared to coprological detection of the *F. hepatica* eggs in feces.

## 4. Discussion

To our knowledge, this is the first meta-analysis based on serodiagnosis of hepatic fasciolosis in humans, cattle, and sheep. Thirty-three studies were included, and a meta-analysis was performed on 27 of these. In general, the studies were of moderate methodological quality and were clinically heterogeneous. All studies analyzed in this meta-analysis used cattle serum samples and confirmed fasciolosis through fecal analysis, which is considered the gold standard test for diagnosing this disease. Overall, *Fh*rCL-1, *Fh*ES, and *Fh*SAP-2 antigens presented the best sensitivity and specificity results for the serum diagnosis of animal and human fasciolosis. The quality of the articles was assessed based on the criteria outlined in QUADAS or STARD. These criteria encompassed the characterization of the samples, the time elapsed between the reference standard and index test, and the demographic characteristics of the animal population under study. After evaluating the results, it was found that none of the articles fulfilled all the QUADAS and STARD criteria.

Fasciolosis is a neglected tropical disease diagnosed through coprology and serological methods (1). A large number of antigens (both native and recombinant) were used in the ELISA tests described in the articles. Among the papers included in the meta-analysis studied the human *Fh*ES antigen group. *Fh*ES has been employed for diagnosing human and bovine fasciolosis in ELISA tests and has proven highly effective (47–50). *Fh*ES antigens play a role in assisting the parasite’s migration through the host tissue. Thus, immunoglobulins for this antigen can be detected in early *F. hepatica* infection (21, 24, 36). Serological tests for the diagnosis of human fasciolosis showed good efficacy when human and animal antibodies for *F. hepatica* were detected using the *Fh*ES antigen in the ELISA tests. However, purification of cysteine proteinase is a complex and time-consuming process (18, 22, 34) that can make the production of a commercial ELISA test difficult.

### 4.1. Native *Fasciola hepatica* antigen

The antigenic preparations used in this study, including the human *Fh*SA protein group, were primarily sourced from adult worm extracts and excretion products, as well as partially purified fractions (51–53). Antibody detection assays were preferred for the immune diagnosis of fasciolosis (27, 29) due to their relative simplicity and early seroconversion during primary infections (3). Since *F. hepatica* is the main cause of human and animal fasciolosis, most of the studies investigating the diagnosis of this disease focused on subunits purified from either *Fh*SA or *Fh*ES antigens specific to this fluke species (27, 51, 54). For the human antigen groups (*Fh*SA and *Fh*ES), only three articles provide a thorough characterization of the study population (46, 47, 52). Most articles that investigate *Fh*SA and *Fh*ES antigens in humans utilize samples from hospitals (23–25, 27, 48–51, 53). As a result, it is challenging to determine the timing of infection, but it is likely that these are chronic infections. Only one article mentions the detection of acute infection by *F. hepatica* (47).

Six studies were included in the meta-analysis evaluating the cattle *Fh*ES protein group. Excretory-secretory products (ESPs) are the antigens that were most commonly used together with ELISA methods for antibody detection. The *Fh*ES antigens of *F. hepatica* used in ELISA tests are known to be immunodominant in cattle naturally exposed to *F. hepatica* infection (3, 35, 57). Native antigens of *F. hepatica* can be collected at bovine abattoirs and used in the laboratory for ELISA tests. This meta-analysis is in line with previous studies, which showed that a cattle *Fh*ES protein group plays a valuable role in an ELISA system for the serodiagnosis of bovine fasciolosis (22, 54, 55). Based on the cattle *Fh*ES antigen group, ELISA tests have been used to detect experimental infections in cattle from the third to the fifth week after infection (22, 35, 56). However, although these studies have good experimental designs, they are limited by the lack of clinical and epidemiological information on the animals. For the cattle *Fh*ES proteins paper group, one article had good sample characterization (22). Articles with natural and experimental infections in cattle were selected. Approximately 100 metacercariae were used in experimental infection studies on cattle (22, 35, 56). In papers with experimental infections, antibody detection occurred between 2 and 4 weeks post-infection (22, 56).

For the *Fh*SA sheep group, only two papers were selected, and a meta-analysis was not performed. These papers reported a sensitivity of 80% and specificity of 90% (59, 60), which was relatively low compared to other antigens (21, 24, 25). Another study that used *Fh*SA as an antigen in an ELISA test reported a sensitivity and specificity of close to 100% (20). The use of the *Fh*SA and *Fh*ES native antigens for routine diagnostic laboratory testing presents some challenges, including the dependence on the availability of living flukes and the fact that it is an antigen mixture subjected to variations due to natural conditions (24, 25). However, laboratories can obtain recombinant antigens, and it has been shown that large quantities of highly pure recombinant *F. hepatica* antigens with correct folding play a vital role in improving the antigenicity and accuracy of serodiagnosis methods (22, 24, 25, 36). For the sheep *Fh*ES proteins, articles were found on both natural and experimental infections. The samples were obtained from farms and abattoirs (57–59). However, none of the studies provided a thorough sample characterization. In the papers with experimental infections, antibody detection occurred between 1 and 3 weeks post-infection (60). Sheep were the hosts where the antibody was identified earliest (36, 60).

### 4.2. Recombinant *Fasciola hepatica* antigen

In terms of human groups using the *Fh*rFtn-1 antigen, just one paper was selected. In this paper, the sensitivity and specificity were close to 100% (61). *Fh*rFtn-1 is expressed during parasite development and has been shown to be highly reactive with sera from experimental animals with acute or chronic infections. However, it is important to highlight that the *Fh*rFtn-1 antigen presented cross-reactivity for other parasites (61), therefore compromising the effectiveness of the ELISA test.

The *Fh*rGST antigen has high antibody titers during active sheep infections, indicating that these molecules are repeatedly and effectively exposed to the host immune system. Cross-reactivity between fasciolosis and echinococcosis can be observed with the *Fh*rGST and *Fh*rFAB antigens used in ELISA tests (28); consistent results and adjustments are necessary for the ELISA test using the *Fh*rGST and *Fh*rFAB antigens for commercialization.

The human group using *Fh*rTP 16.5, a small antigen of the tegument of *F. hepatica* expressed in bacteria, showed sensitivity and specificity that were close to 90% (61). The tegumental surface of *F. hepatica* is a unique syncytial structure that serves as an interface between the parasite and host. The *Fh*rTP antigens are easily released to stimulate the host immune response and are therefore considered diagnostic antigens (3, 61). The *Fh*rTP antigen is located on the parasite’s surface and has cross-reactivity with other parasites (62), compromising the quality of ELISA tests. Furthermore, current parasitological methods depend on the technician’s expertise, as *F. hepatica* eggs can be confused with those of other helminths (4, 13).

Among the subunit antigens, cathepsin-L, a component of *Fasciola* ES antigens, garnered significant attention. Serological tests have shown that they are highly accurate in diagnosing human, cattle, and sheep fasciolosis. The recombinant cathepsin L1 test uses recombinant pro-cathepsin L1 and targets antibodies against cathepsin, a cysteine protease, to diagnose fasciolosis caused by *F. hepatica*. Similarly, other studies have not found cross-reactions in cathepsin-based ELISA tests (21) and have reported good performance. The ELISA test yielded better results with *Fh*ES, a native antigen collected from *F. hepatica* obtained in a bovine abattoir. The second most important antigen used in ELISA tests was *Fh*rCL-1, a recombinant antigen expressed in bacteria and yeast. One article had good sample characterization for the cattle *Fh*rCL-1 protein paper group (22). In the experimental infection in cattle, antibodies against *F. hepatica* were identified 3 weeks post-infection (22, 36).

Only two papers were selected for human groups using *Fh*rSAP-2, and it was therefore impossible to conduct a meta-analysis. These antigens are expressed in *E. coli* (24, 25). The papers that used the *Fh*rSAP-2 antigen for serum diagnosis of *F. hepatica* in humans showed a sensitivity of 100% and a specificity higher than 95% (24, 25). Previous studies have also shown that *Fh*rSAP-2 is highly immunogenic and can detect the acute phase of fasciolosis (24, 25, 28). In sheep *Fh*rCL-1, two studies were selected, and no meta-analysis was performed. The sensitivity and specificity were very high for *Fh*rCL-1 (28, 29). Analysis of different cloning and variations of purification methods has shown diverse levels of sensitivity, specificity, and accuracy in diagnostic tests. For the last human group using *Fh*rCL-1, just two papers were selected, and a meta-analysis was not carried out (21, 24); in these articles, the sensitivity and specificity were close to 100%. The *Fh*rCL-1 antigen is localized in excretory and secretory proteins and has no cross-reactivity with other parasites (21, 24). A good diagnostic test must distinguish between *F. hepatica* and other parasitic diseases.

Nine of the 33 studies analyzed used recombinant antigens in the ELISA test. *Fh*rCL-1, *Fh*ES, and *Fh*rSAP-2 antigens gave the best results, with high sensitivity and specificity values for fasciolosis serodiagnosis in humans and animals. The recombinant antigen can be used in ELISA tests for non-invasive or bulk tank milk samples for epidemiological studies. Serological studies are now the main diagnostic method in use, enabling disease diagnosis even during the acute stage and before the parasite eggs can be identified in feces. Serology has the advantage of identifying infections much earlier than fecal egg identification (around 4–5 weeks) (21, 25, 29). The serological methods, especially the ELISA test, are highly sensitive and specific compared to diagnosing *F. hepatica* by coprological methods (25, 27, 29). Recombinant proteins allow for increased mass screening, facilitating fasciolosis serodiagnosis in humans and animals.

Our meta-analysis has shown that early antibodies against *F. hepatica* can be detected in both animals and humans. This early detection is made possible through the use of native and recombinant antigens in ELISA tests (21, 22, 51, 54, 58). However, the studies included in the meta-analysis did not adequately distinguish between acute and chronic fasciolosis infections. Despite this, the results obtained indicated high sensitivity and specificity values for various antigens in both animals and humans. By employing these antigens in ELISA tests, it becomes possible to accurately identify *F. hepatica* antibodies, thereby reducing the occurrence of false positives or false negatives. Nevertheless, despite the promising findings from the meta-analysis, the availability of antigens in the form of ELISA tests for the systematic identification of fasciolosis in animals and humans remains limited (36, 47, 49). Currently, only a small number of native and recombinant antigens are commercially accessible in the form of ELISA tests for widespread use (22, 47, 51, 52).

Our study has some limitations that need to be addressed. Firstly, the number of studies included in the meta-analysis is relatively small, which restricts our ability to conduct relevant subgroup analyses, such as age and time of infection. This limitation is caused by the lack of consistent data found in the available literature. However, this limitation highlights the importance of further research in the literature to gather more data, aiming to provide high-quality scientific evidence for the incorporation of these tests in disease screening and early diagnosis. Despite these limitations, it is crucial to acknowledge the robustness and low heterogeneity of the data obtained in our study. These factors have allowed us to draw sound conclusions from the results we have obtained so far. Future research with a larger and more diverse pool of studies will be valuable to expand and corroborate our findings.

## 5. Conclusion

The meta-analysis results showed eight antigen types for serum diagnosis of fasciolosis in humans, cattle and sheep. Most articles analyzed used ES antigens in humans. It is therefore suggested that *Fh*rCL-1, *Fh*ES, and *Fh*rSAP-2 could be considered ideal diagnostic antigens for the earliest serum diagnosis of human and animal fasciolosis. We recommend future studies with *F. hepatica* antigens for serological diagnosis in other animal species and the need for the literature to include more robust and well-characterized studies.

## Author contributions

GD: conceptualization, methodology, and manuscript writing. TV: conceptualization, methodology, manuscript writing and reviewing. VB: data curation, meta-analysis, and manuscript reviewing. MP: meta-analysis and manuscript reviewing. JR and LM: manuscript reviewing. FF: manuscript writing, reviewing, and editing. All authors contributed to the article and approved the submitted version.

## Conflict of interest

The authors declare that the research was conducted in the absence of any commercial or financial relationships that could be construed as a potential conflict of interest.

## Publisher’s note

All claims expressed in this article are solely those of the authors and do not necessarily represent those of their affiliated organizations, or those of the publisher, the editors and the reviewers. Any product that may be evaluated in this article, or claim that may be made by its manufacturer, is not guaranteed or endorsed by the publisher.
