# Supplementary material for: Serological diagnosis of fasciolosis (Fasciola hepatica) in humans, cattle, and sheep: a meta-analysis
Source: Front Vet Sci. 2023 Aug 31;10:1252454. doi: 10.3389/fvets.2023.1252454 (PMC10509555; doi:10.3389/fvets.2023.1252454)
Supplement: Supplementary file 1 [file Data_Sheet_1.pdf]

Chart 1: List of papers with each assessment of quality results by QUADAS criteria.

	Criteria								Score		
**Papers**	**1**	**2**	**3**	**4**	**5**	**6**	**7**	**8**	**Less to 4 criteria**	**4 to 6 criteria**	**7 or more**
Cabán-Hernández et al 2013	Yes	Yes	No	Yes	Yes	Yes	Yes	Unclear		Good	
Carnevele et al 2001	Yes	Yes	Unclear	Yes	Yes	Yes	Yes	Unclear		Good	
Cordova et al 1999	Yes	Yes	Unclear	Unclear	Yes	Yes	Yes	Unclear		Good	
Cornelissen et al 1992	Yes	Yes	Yes	Yes	Yes	Yes	Yes	Unclear			Optimal
Cornelissen et al 1999	Yes	Yes	Yes	Yes	Yes	Yes	Yes	Unclear			Optimal
Cornelissen et al 2001	Yes	Yes	Yes	Yes	Yes	Yes	Yes	Unclear			Optimal
El-Shazly et al 2002	Yes	Yes	Unclear	Yes	Yes	Unclear	Yes	Unclear		Good	
Espinoza et al 2005	Yes	Yes	Unclear	Unclear	Yes	No	Yes	Unclear		Good	
Espinoza et al 2007	Yes	Yes	Yes	Yes	Yes	Yes	Yes	Unclear			Optimal
Figueroa-Santiago et al 2011	Yes	Yes	Yes	Yes	Yes	Yes	Yes	Unclear			Optimal
Gottstein et al 2014	Yes	Yes	Unclear	No	Yes	Yes	Yes	Unclear		Good	
Heidari et al 2015	Yes	Yes	Unclear	Yes	Yes	Yes	Yes	Unclear		Good	
Hillyer et al 1996	Yes	Yes	Yes	Yes	Yes	Yes	Yes	Unclear			Optimal
Ikeda 1998	Yes	Yes	Unclear	No	Yes	Yes	Yes	Unclear		Good	
Kazantseva et al 2017	Yes	Yes	Unclear	Yes	Yes	Yes	Yes	Unclear		Good	
Kooshan et al 2010	Yes	Yes	Yes	No	Yes	Yes	Yes	Unclear		Good	
Kuerpick et al 2013	Yes	Yes	Yes	Unclear	Yes	Yes	Yes	Yes			Optimal
Maher et al 1999	Yes	Yes	Yes	Unclear	Yes	Yes	Yes	Unclear		Good	
Martinez-Sernandez et al 2018	Yes	Yes	Yes	Yes	Yes	Yes	Yes	Unclear			Optimal
Mezo et al 2003	Yes	Yes	Yes	No	Yes	Yes	Yes	Unclear		Good	
Mirzadeh et al 2018	Yes	Yes	Unclear	Yes	Yes	Yes	Yes	Unclear		Good	
Mokhtarian et al 2018	Yes	Yes	Yes	Yes	Yes	Yes	Yes	Unclear			Optimal
Morales & Espino 2012	Yes	Yes	Yes	Yes	Yes	Yes	Yes	Unclear			Optimal
Mufti et al 2015	Yes	No	Yes	Yes	Yes	Yes	Yes	Unclear		Good	
Rahini et al 2011	Yes	Yes	Yes	Unclear	Yes	Yes	Yes	Unclear		Good	
Rokni et al 2002	Yes	Yes	Yes	Unclear	Yes	Yes	Yes	Unclear		Good	
Salimi-bejestani et al 2005	Yes	Yes	Yes	Yes	Yes	Yes	Yes	Unclear			Optimal
Santana et al 2013	Yes	Yes	Unclear	Unclear	Yes	Yes	Yes	No		Good	
Shafiei et al 2015	Yes	Yes	Yes	Unclear	Yes	Yes	Yes	Unclear		Good	
Simsek et al 2006	Yes	Yes	Yes	Unclear	Yes	Yes	Yes	Unclear		Good	
Aguayo et al 2018*	Yes	Yes	Yes	Yes	Yes	Yes	Yes	Unclear			Optimal

Cornejo et al 2010*	Yes	Yes	Yes	Yes	Yes	No	Yes	Unclear		Good
Mirzadeh et al 2017*	Yes	Yes	Unclear	Unclear	Unclear	No	Yes	Unclear	Regular	

*Pubmed database.

Chart 2: List of papers with each assessment of quality results by STARD criteria.

	Criteria			Score	
**Paper**	**1**	**2**	**3**	**To 2 criteria**	**More to 3**
Cabán-Hernández et al 2013	Yes	Yes	No	Regular	
Carnevele et al 2001	Yes	No	No	Regular	
Cordova et al 1999	Yes	No	No	Regular	
Cornelissen et al 1992	Yes	No	Yes	Regular	
Cornelissen et al 1999	Yes	No	Yes	Regular	
Cornelissen et al 2001	Yes	No	Yes	Regular	
El-Shazly et al 2002	Yes	No	Yes	Regular	
Espinoza et al 2005	Yes	No	No	Regular	
Espinoza et al 2007	Yes	No	Yes	Regular	
Figueroa-Santiago et al 2011	Yes	No	No	Regular	
Gottstein et al 2014	Yes	No	No	Regular	
Heidari et al 2015	Yes	No	No	Regular	
Hillyer et al 1996	Yes	No	No	Regular	
Ikeda 1998	Yes	No	No	Regular	
Kazantseva et al 2017	Yes	Yes	No	Regular	
Kooshan et al 2010	Yes	No	No	Regular	
Kuerpick et al 2013	Yes	Yes	Yes		Optimal
Maher et al 1999	Yes	No	Yes	Regular	
Martinez-Sernandez et al 2018	Yes	No	Yes	Regular	
Mezo et al 2003	Yes	No	Yes	Regular	
Mirzadeh et al 2018	Yes	No	No	Regular	
Mokhtarian et al 2018	Yes	No	No	Regular	
Morales & Espino 2012	Yes	No	No	Regular	
Mufti et al 2015	Yes	No	Yes	Regular	
Rahimi et al 2011	Yes	No	No	Regular	
Rokni et al 2002	Yes	No	Yes	Regular	
Salimi-bejestani et al 2005	Yes	Yes	Yes		Optimal
Santana et al 2013	Yes	No	Yes	Regular	
Shafiei et al 2015	Yes	Yes	No	Regular	
Simsek et al 2006	Yes	No	No	Regular	
Aguayo et al 2018*	Yes	No	No	Regular	

Cornejo et al 2010*	Yes	Yes	No	Regular
Mirzadeh et al 2017*	Yes	No	No	Regular

* Pubmed database

Chart 3. Characteristics of studies analyzed in meta-analysis

Author	Specie	Participant Characteristics	Clinical Setting	Study Design	Target Condition Definition	Index Test	Reference Standard	Sample Size	Funding Sources
Carnevale et al 2001	Human	Naturally infection	Not clear	Validation diagnostic test	Secretory/excretory antigens	ELISA	Coprological	341 samples (22 positive, 100 negative, and 219 other parasites)	No Mentioned
Cordova et al 1999	Human	Naturally infection	Not clear	Validation diagnostic test	Secretory/excretory antigens	ELISA	Coprological and imunoeletroforese	138 samples (38 positive, 46 negative and 54 other parasites)	No Mentioned
Cornelissen et al 1999	Bovine	Naturally and experimental infection	Farm	Validation diagnostic test	Secretory/excretory antigens	ELISA	Coprological and macroscopic inspection of the liver	421 samples (95 positive, 183 other parasites and negative)	No Mentioned
Cornelissen et al 2001	Ovine and bovine	Naturally and experimental infection	Farm	Validation diagnostic test	Recombinant cathepsin	ELISA	Coprological	204 ovine (31 positive and 119 other parasites) and 568 bovine (106 positive and 332 other parasites) and negative	No Mentioned
Espinoza et al 2005	Human	Naturally infection	Not clear	Validation diagnostic test	Secretory/excretory antigens	ELISA	Coprological	138 samples (38 positive, 46 negative and 54 other parasites)	Yes
Espinoza et al 2007	Human	Naturally infection	Not clear	Validation diagnostic test	Secretory/excretory antigens	ELISA	Coprological	634 feces and 613 serum (46 negative and other parasites by stool examination)	Yes
Figueroa-Santiago et al 2011	Human	Naturally infection	Sample bank	Validation diagnostic test	Secretory/excretory antigens	ELISA	Coprological	127 samples (37 positive, 50 negative and 40 other parasites)	Yes
Gottstein et al 2014	Human	Naturally infection	Not clear	Validation diagnostic test	Secretory/excretory antigens, recombinant saposin and recombinant cathepsin	ELISA	Coprological	257 samples (30 positive, 20 negative, 87 other parasites and 121 cancer)	No Mentioned
Heidari et al 2015	Ovine	Experimental infection	Abattoir	Validation diagnostic test	Secretory/excretory antigens	ELISA	Coprological and macroscopic inspection of the liver	281 samples (50 positive, 150 negative and 81 other parasites)	No

Hillyer et al 1996	Ovine and bovine	Naturally and experimental infection	Farm	Validation diagnostic test	Secretory/excretory antigens	ELISA	Coprological	184 ovine (22 positive) and 147 bovine (38 positive) and negative	Yes
Kooshan et al 2010	Ovine	Naturally infection	Abattoir	Validation diagnostic test	Secretory/excretory antigens and Somatic antigen	ELISA	Coprological and macroscopic inspection of the liver	140 samples (30 positive, 10 negative and 100 for validation)	Yes
Kuerpick et al 2013	Bovine	Experimental infection	Farm	Validation diagnostic test	Secretory/excretory antigens and recombinant cathepsin	ELISA	Coprological and Sorological	140 samples (20 positive, 100 negative and 20 other parasites)	Yes
Maher et al 1999	Human	Naturally infection	Hospital	Validation diagnostic test	Somatic antigen	ELISA	Coprological	176 samples (60 positive, 20 negative and 96 other parasites)	No Mentioned
Martinez-Sernandez et al 2018	Ovine and bovine	Naturally infection	Abattoir	Validation diagnostic test	Recombinant cathepsin	ELISA	Coprological and Sorological	300 samples (100 positive, 200 negative and other parasites)	Yes
Mezo et al 2003	Ovine	Naturally infection	Abattoir	Validation diagnostic test	Secretory/excretory antigens	ELISA	Coprological and macroscopic inspection of the liver	142 samples (46 positive, 50 negative and 46 other parasites)	Yes
Mokhtarian et al 2018	Ovine	Naturally infection	Abattoir	Validation diagnostic test	Secretory/excretory antigens, recombinant fatty acid binding protein, recombinant glutathione S-transferase and recombinant cathepsin	ELISA	Coprological and macroscopic inspection of the liver	113 samples (30 positive, 40 negative and 43 other parasites)	Yes
Morales & Espino 2012	Human	Naturally infection	Hospital	Validation diagnostic test	Secretory/excretory antigens	ELISA	Coprological	119 samples (45 positive, 33 negative and 41 other parasites)	Yes
Mufti et al 2015	Bovine	Experimental infection	Farm	Validation diagnostic test	Secretory/excretory antigens	ELISA	Coprological	200 samples	No Mentioned
Rahimi et al 2011	Human	Naturally infection	Not clear	Validation diagnostic test	Somatic antigen	ELISA	Coprological ELISA, Immunofluorescence and surgical confirmations	84 samples (35 positive, 22 negative and 27 other parasites)	Yes

Rokni et al 2002	Human	Naturally infection	Not clear	Validation diagnostic test	Secretory/excretory antigens	ELISA	Coprological	268 samples (176 positive, 15 negative and 77 other parasites)	No Mentioned
Salimi-bejestani et al 2005	Bovine	Experimental infection	Farm	Validation diagnostic test	Secretory/excretory antigens	ELISA	Coprological	526 samples (264 positive, 256 negative and 6 other parasites)	No Mentioned
Santana et al 2013	Human	Naturally infection	Hospital	Validation diagnostic test	Recombinant cathepsin	ELISA	Coprological	386 samples (93 positive and 135 negative and 158 other parasites)	Yes
Shafiei et al 2015	Human	Naturally infection	Not clear	Validation diagnostic test	Somatic antigen	ELISA	Coprological	77 samples (15 positive and 30 negative and 32 other parasites)	Yes
Simsek et al 2006	Bovine	Naturally infection	Farm	Validation diagnostic test	Secretory/excretory antigens	ELISA	Coprological and macroscopic inspection of the liver	1200 samples	No Mentioned
Aguayo et al 2018	Human	Naturally infection	Not clear	Validation diagnostic test	Secretory/excretory antigens	ELISA	Coprological	116 samples (36 positive, 36 negative and 44 other parasites)	Yes
Cornejo et al 2010	Human	Naturally infection	Not clear	Validation diagnostic test	Secretory/excretory antigens	ELISA	Coprological	298 samples (33 positive, 177 negative and 88 other parasites)	Yes
Mirzadeh et al 2017	Human	Naturally infection	Not clear	Validation diagnostic test	Secretory/excretory antigens	ELISA	Coprological	201 samples (52 positive, 70 negative and 79 other parasites)	Yes
